# Enhanced rehabilitation after total joint replacement using a wearable high-density surface electromyography system

**DOI:** 10.3389/fresc.2025.1657543

**Published:** 2025-10-16

**Authors:** Richard Morsch, Tim Böckenförde, Milan Wolf, Stefan Landgraeber, Daniel J. Strauss

**Affiliations:** ^1^ Systems Neuroscience & Neurotechnology Unit, Medical Faculty, Saarland University, Homburg, Germany; ^2^ Medical Faculty, Saarland University, Saarbrücken, Germany; ^3^ Center for Digital Neurotechnologies Saar, Homburg, Germany; ^4^ Clinics for Orthopedics and Orthopedic Surgery, Saarland University Medical Center, Homburg, Germany

**Keywords:** HD-sEMG, electromyography (EMG), rehabilitation, TJA, wearable EMG system, neuromuscular recovery, rehabilitation monitoring, functional indices

## Abstract

**Introduction:**

Neuromuscular recovery after total joint arthroplasty remains insufficiently understood, and current tools for assessing muscle function lack the resolution to monitor detailed recovery dynamics. High-Density surface Electromyography (HD-sEMG) enables spatiotemporal analysis of muscle activation and may support individualized rehabilitation. However, its clinical application in orthopedic settings remains limited.

**Methods:**

This exploratory study presents a methodological framework for applying wearable 64-channel HD-sEMG system to monitor neuromuscular recovery in patients undergoing total knee or hip arthroplasty. HD-sEMG data were recorded during standardized mobilization exercises at multiple pre- and postoperative time points. A custom signal processing pipeline was developed, encompassing artifact suppression, dimensionality reduction, feature extraction, and the derivation of five functional indices summarizing key aspects of muscle performance.

**Results:**

Initial clinical application demonstrated the feasibility of the approach. The functional indices revealed distinct recovery dynamics across patients and showed promising alignment with patient-reported outcome measures. Individual case analyses suggested the potential of HD-sEMG to differentiate between restitution and dysfunctional compensation patterns.

**Discussion:**

This study provides a structured, exploratory foundation for longitudinal HD-sEMG research in orthopedic rehabilitation. While not yet suited for clinical decision-making, the proposed framework offers methodological tools for future investigations of neuromuscular recovery trajectories and may contribute to the development of personalized, data-driven rehabilitation strategies.

## 1 Introduction

### 1.1 Clinical background and motivation

Recovery after total joint arthroplasty (TJA) varies greatly between individuals, and the underlying factors influencing this variability remain insufficiently understood. General muscle weakness is associated with increased surgical risk and poor functional outcomes (1, 2). However, the specific mechanisms through which muscular condition impacts recovery trajectories have yet to be clarified (3).

There is evidence of postoperative neuromuscular alterations following total knee (TKA) and hip arthroplasty (THA), including reduced quadriceps activation, asymmetrical recruitment patterns, and persistent gluteal muscle dysfunction, implying deficits in neural drive and the need for targeted rehabilitation (3, 4). Suboptimal rehabilitation can further diminish treatment outcomes and patient satisfaction, despite technically successful surgery (5–8).

### 1.2 High-density surface electromyography (HD-sEMG), a promising tool for objective monitoring

In recent years, HD-sEMG has emerged as a powerful tool for analyzing spatial and temporal patterns of myoelectric activity. Its two-dimensional electrode-array arrangement allows for a detailed spatiotemporal analysis of muscle activation and motor unit behavior, offering advantages over conventional bipolar EMG and invasive intramuscular techniques (9–12). Furthermore, technical innovations have made HD-sEMG systems more accessible and easier to implement in clinical practice. Recent technological advances like wearable systems, improved electrode materials, and robust decomposition algorithms have increased its usability and accuracy in dynamic, real-world settings (13, 14).

### 1.3 Clinical relevance and limitations

HD-sEMG has been systematically applied across a range of neuromuscular disorders, demonstrating diagnostic value in conditions such as motor neuron diseases, neuropathies, myopathies, and muscle fatigue assessment (15). Its non-invasive nature makes it particularly suitable for populations where needle electromyography is impractical, such as pediatric or longitudinal applications, while enabling detection of pathological motor unit changes (9). HD-sEMG also provides complementary biomarkers to conventional diagnostics, enhancing clinical evaluation of neuromuscular function (16). Despite its diagnostic potential and remarkable advances, HD-sEMG still faces several challenges for broader clinical adoption, including variability in electrode placement, standardization of signal processing pipelines, and the need for normative reference data across populations (17).

### 1.4 State of the art: a research gap in total joint arthroplasty

Despite the widespread use of surface EMG in TJA research (3, 18, 19) to name a few, HD-sEMG remains underrepresented in this context. No published longitudinal HD-sEMG studies exist for TKA or THA patients to date. Existing research primarily consists of single-time-point or methodological studies, often in healthy cohorts (20, 21). This highlights the need for longitudinal, clinically embedded HD-sEMG investigations.

### 1.5 Study aim

This study bridges modern neurotechnology and orthopedic rehabilitation to enhance the objectivity, treatment individualization, and efficiency of recovery. It presents a methodological framework for applying wearable HD-sEMG to monitor neuromuscular recovery after TKA and THA. A wearable 64-channel HD-sEMG system was employed to capture myoelectric activity in patients undergoing the TJA under real-world conditions. Studies of both TKA and THA demonstrate significant intraoperative and perioperative practice variability among high-volume surgeons, including differences in surgical approach, implant type, use of tourniquet, patellar resurfacing, closure technique, and perioperative medications (22, 23). However, TJA procedures at the study's clinical site are performed according to a highly standardized protocol, ensuring comparability across patients. Measurements were conducted at multiple time points pre- and post-surgery, while patients performed a standardized set of mobilization exercises supervised by a physiotherapist. This paper proposes a signal processing pipeline in order to extract key spatial, spectral, and temporal features relevant to clinical recovery monitoring. It outlines the feasibility, challenges, and diagnostic potential of HD-sEMG-based rehabilitation monitoring in a real-world clinical setting.

## 2 Method

**Figure 1:**
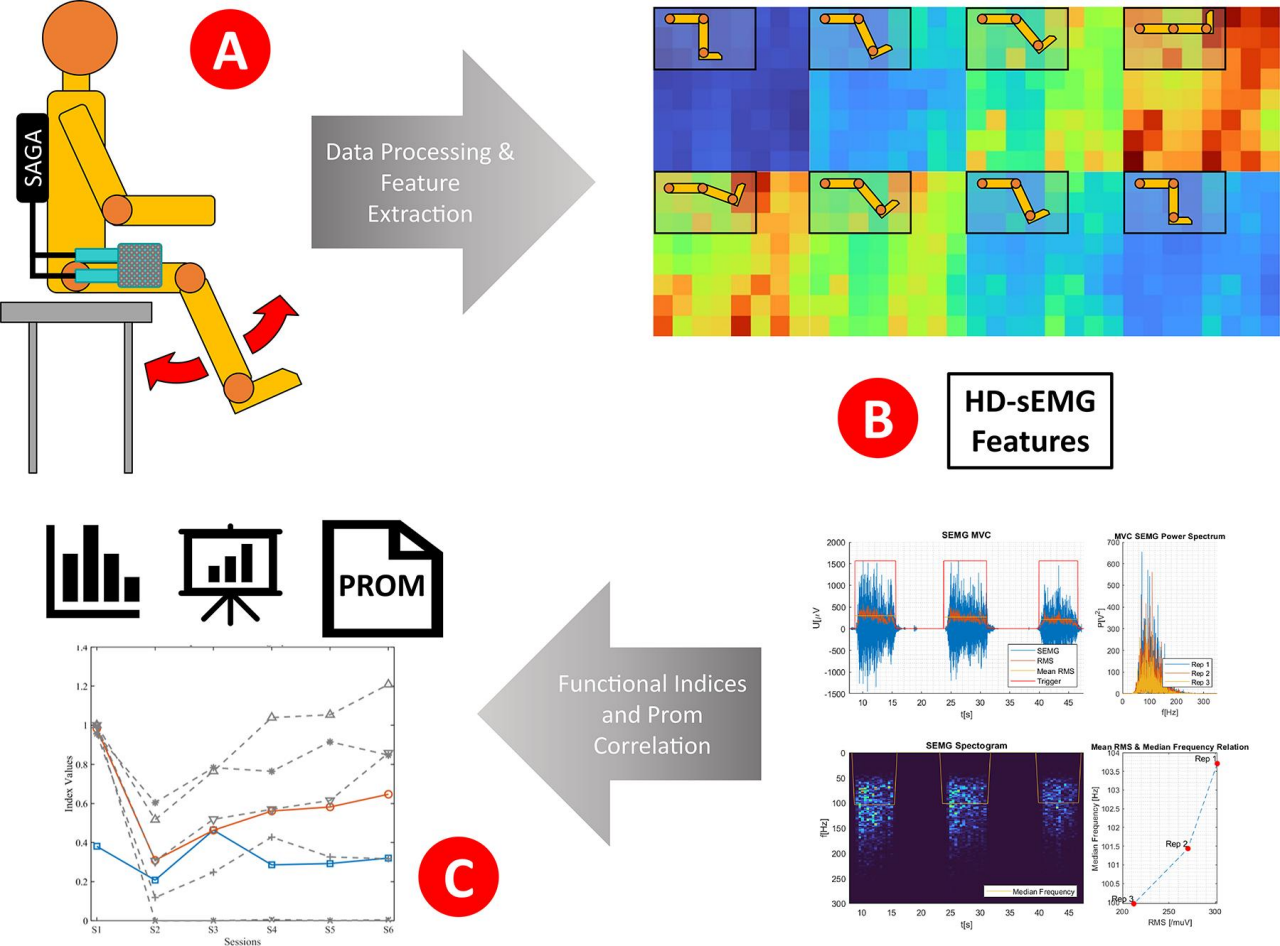
Conceptual overview of the HD-sEMG assessment and analysis workflow. HD-sEMG signals were recorded during functional leg movements using a wearable textile grid and the TMSi SAGA system **(A)**. The EMG signals were processed to extract spatial, temporal, and spectral features and combined into five functional indices. Simultaneously, PROMs were collected across multiple postoperative sessions **(B)**. Indices and PROM scores were then analyzed and compared to assess neuromuscular recovery and functional progression over time **(C)**.

Figure 1 provides a schematic overview of the measurement design and the subsequent analysis workflow.

### 2.1 Study design and setting

This prospective observational study was designed to identify muscle-level factors, potentially predictive, influencing postoperative outcomes and patient satisfaction following TKA or THA. Measurements were conducted at Clinics for Orthopedics and Orthopedic Surgery, Saarland University Medical Center, Homburg (Saar), Germany. The study was approved by the Ethics Committee of the Medical Association of Saarland (approval number: HA273/20).

### 2.2 Study population

A total of 63 patients (34 M, 29 W; mean age ∼64 ± 10 years; 33 TKA and 30 THA) participated in the study. In all cases, the diagnosis was knee or hip osteoarthrosis. For this report, three exemplary patients were selected from the study population to illustrate initial trends and highlight the interpretive potential of the proposed methods. The selected cases include patient 016 (65 years, male; diagnosis: gonarthrosis; surgery: medial parapatellar approach; implant system: Persona®, Zimmer Biomet), 018 (75 years male; diagnosis: gonarthrosis; surgery: medial approach; implant system: Persona®, Zimmer Biomet) and 031 (68 years, male; diagnosis: coxarthrosis; surgery: lateral approach; implant system: AESCULAP® CoreHip®, B. Braun SE).

Patients were recruited at the day of preoperative assessment. After receiving detailed study information and providing written consent, they were enrolled in the study. Patients undergoing primary total knee or hip arthroplasty were included without restrictions on BMI or age. Eligibility required the ability to independently perform the measurement exercises and complete questionnaires, allowing for both pre- and postoperative HD-sEMG and PROMs data collection. Exclusion criteria included lack of informed consent, minor status, neurological or metabolic muscle disorders potentially affecting muscle activity, and incomplete datasets or missing follow-up assessments.

### 2.3 Measurement setup

#### 2.3.1 Measurement workflow

PROMs were acquired at the beginning of each measurement session. Under supervision, the patients then performed the mobilization exercises while HD-sEMG was recorded. If feasible for the patient, both the operated and contralateral sides were assessed.

Measurements were conducted ∼5 days pre-surgery and up to five times post-surgery (∼2 days, 4–5 days, 6 weeks, 3 months, and 6 months).

#### 2.3.2 Materials

HD-sEMG was recorded using the TMSi SAGA system (High Density Amplifier and 64-channel Ag/AgCl HD-EMG Grids; Artinis Medical Systems, Netherlands). Patient-Reported Outcome Measures (PROMs), either knee injury and osteoarthritis outcome score (KOOS) or hip disability and osteoarthritis outcome score (HOOS) served as clinically accepted measures (24, 25).

#### 2.3.3 Electrode placement

Electrodes were positioned at *m. rectus femoris* (TKA) and *m. gluteus medius* (THA). The electrode sites were prepared by cleaning the skin with disinfectant, applying a mild abrasive gel, and using conductive electrode gel to minimize contact impedance and ensure optimal signal quality. In order to ensure consistent electrode placement and to minimize crosstalk this was done according to the SENIAM guidelines (surface EMG for a non-invasive assessment of muscles) and anatomical landmarks proposed by Hermens et al. for standardized and reproducible sEMG recording (26).

#### 2.3.4 Exercise protocol

Two different mobilization exercise routines were used for TKA and THA patients, respectively. These exercises are well-established components of the clinic's standard rehabilitation protocol. Patients were encouraged by the supervising therapist to perform ideally ten or more repetitions per exercise and side; however, depending on individual physical condition or subjective pain levels, this could not always be achieved. The routines consisted of eight different exercises divided into two parts: Part one was performed while lying or sitting on the examination table, and part two in a standing position with optional handrail support.

The exercises for TKA included: a) foot dorsiflexion and plantarflexion in a lying position, b) knee flexion and extension with the foot sliding on the examination table, c) maximal isometric knee extension by pressing the straight leg into the examination table while lying down, d) knee flexion and extension with the foot elevated, e) knee flexion and extension while sitting upright on the examination table, f) rolling motion: upright standing in a narrow lunge position with the operated leg positioned slightly behind, performing a step-like rolling movement with the operated foot, g) loading: forward lunge with the operated leg in front, actively shifting bodyweight onto it, h) posterior stretching: backward lunge with the operated leg extended behind, shifting bodyweight onto the front leg to stretch the posterior chain.

The exercises for THA included. a) foot dorsiflexion and plantarflexion in a lying position, b) hip flexion and extension with the foot sliding on the examination table, c) hip flexion and extension with the foot elevated, d) hip abduction in a lying position, e) maximal isometric hip extension by pressing the straight leg into the examination table while lying down, f) hip flexion while standing upright by lifting the bent leg, g) hip abduction with the extended leg while standing upright, h) hip extension while standing upright by moving the straight leg posteriorly.

### 2.4 Signal processing

#### 2.4.1 Data acquisition and initial inspection

HD-sEMG was recorded at a sampling rate of 1,000 Hz using the TMSi SAGA system. A manual trigger signal was acquired synchronously to the EMG data via the same system to indicate the currently performed exercise and its respective repetition (i.e., motion onset and peak). Following data acquisition, an initial manual inspection of the raw data was conducted to assess signal quality. Here, manual correction of trigger events enabled precise identification of EMG onset and offset down to the millisecond, which was crucial in this exploratory setting given the variability in patient performance during the exercises. In cases where electrode disconnection or severe motion artifacts were identified during a repetition, the affected trial was marked to be excluded from further analysis. All subsequent processing was performed using a custom MATLAB-based routine.

#### 2.4.2 Data conditioning

##### 2.4.2.1 Pre-processing

The raw EMG signals were bandpass-filtered within a physiological range of 20–450 Hz using sixth-order Butterworth filters. To reduce low-frequency noise such as motion artifacts and DC drift, a high-pass filter at 20 Hz was applied, while high-frequency noise was attenuated by a low-pass filter at 450 Hz (27). Additionally, a second-order IIR notch filter at 50 Hz with a quality factor of 50 was used to suppress power line interference originating from ground loops and electromagnetic noise (28).

##### 2.4.2.2 Channel quality assessment

Channel quality was evaluated based on several criteria: a) Channel disconnects, defined as channels showing no data. b) Insufficient electrode adhesion, indicated by abnormally low signal amplitude. c) Signal-to-noise ratio, estimated as the ratio between power within the physiological frequency band (20–450 Hz) and the signal's total spectral power (27, 29).

Channels meeting one or more of these criteria were flagged as poor quality. If the number of bad channels exceeded two, they were excluded from further processing. Otherwise, affected channels were reconstructed using two-dimensional bilinear interpolation based on neighboring electrodes within the grid.

##### 2.4.2.3 Segmenting

The recorded EMG data were segmented into individual repetitions based on manually validated trigger indices. Each trigger marked the onset of a specific exercise repetition, and segments were extracted from the onset of one repetition to the onset of the subsequent one. This approach ensured that each segment captured a full contraction cycle, independent of temporal variation across repetitions or participants. As movement speed may vary between repetitions, the resulting window lengths were inherently variable and adapted to the actual shape of the recorded activation pattern.

##### 3.4.2.4 Signal quality enhancement

To further improve signal quality and suppress potential residual artifacts (e.g., crosstalk), blind source separation was performed via independent component analysis (ICA), using the MATLAB FastICA algorithm on each segmented repetition (10, 30–32).

Each component was evaluated based on multiple physiologically motivated criteria: spatial focus was assessed by the variance of the associated mixing vector, spectral plausibility required at least 90% of power within the 20–450 Hz band, temporal shape fidelity was evaluated by correlating rectified and smoothed (500 ms windows) components with the repetition-averaged activation profile, while also components with exceptionally low zero crossing rate, low Hoyer's sparseness in either in the component or its mixing vector, or elevated kurtosis were also considered non-physiological (31–34).

Rejected components were excluded from the inverse projection step, resulting in a denoised reconstruction of the EMG signal. The rejection logic could be flexibly adjusted to suit analysis needs—for instance, by excluding all components failing a critical criterion, or by applying thresholds requiring multiple failed metrics. This approach enabled effective noise suppression while maintaining physiologically relevant information.

#### 2.4.3 Feature extraction

A set of temporal, spectral and spatial features was extracted for each segmented repetition in order to characterize the neuromuscular activation pattern from multiple perspectives.

Time-domain features included the integrated EMG (iEMG), root mean square (RMS), and the highest exercise-specific RMS-amplitude as maximum voluntary contraction (MVC) (35). In addition, the activation ratio (AR) was computed as the percentage of time the RMS exceeded defined thresholds (5%–30% MVC), indicating active contraction periods (36). Lastly, sample entropy for each channel was calculated (37).

Frequency-domain features comprised the median frequency (MF), zero crossing rate (ZCR), and band power (BP) distribution within 20–450 Hz, allowing assessment of spectral content, noise, and fatigue indicators (38–40).

Spatial activation was characterized using spatial RMS maps, from which Hoyer's sparseness, center of gravity (CoG), spatial dispersion (Spread), spatial entropy, and max-to-mean ratio (MMR) were derived (41–45).

#### 2.4.4 Functional indices

To facilitate exploratory interpretation of multidimensional EMG features, five functional indices were defined to represent higher-level neuromuscular performance domains. All indices were calculated by combining physiologically related, normalized feature sets.

• Activation Intensity Index (AII): Captures the overall myoelectric activation level, based on iEMG, RMS, BP, and mean AR from 10% to 30%
• Efficiency and Focus Index (EFI): Quantifies spatial activation economy and localization, using, Hoyer's sparseness, MMR, spatial spread (inversely weighted), and spatial entropy.
• Fatigue and Performance Index (FPI): Reflects performance changes across repetitions by evaluating the trend (slope) of activation features (iEMG, RMS, MVC, BP, AR) and control-related features (MF, ZCR, Hoyer's sparseness).
• Coordination and Stability Index (CSI): Represents consistency of neuromuscular control, combining inter-repetition variance of RMS, MF, BP, and CoG.
• Spatial Correlation Index (SCI): Assesses similarity between spatial activation patterns (RMS, BP, MF two-dimensional maps) and a reference template (either mean preoperative or contralateral state), based on correlation across repetitions.

These indices were designed to summarize complex EMG activation behavior into interpretable domains and to support exploratory group comparisons, temporal progression analysis, and correlation with functional recovery markers. For all indices, higher values reflect “better” results compared to reference.

##### 2.4.4.1 Signal variability compensation

To account for interindividual variability and diffuseness of muscle activation, all spatially aggregated features (e.g., iEMG, RMS, BP) were computed over a variable focus area, rather than using a fixed set of channels. Specifically, channels were included if they fell within one weighted standard deviation (i.e., spatial spread) around the CoG of the activation map. This adaptive method aimed for characterization of the most physiologically relevant activation zone, while compensating for inter-session variability in spatial spread or underlying activation patterns. To our knowledge, this aspect has not yet been standardized in current HD-sEMG literature.

##### 2.4.4.2 Index normalization

Towards inter-session and inter-subject comparability, two reference-based versions were generated, for functional indices: one normalized to the preoperative state, and one to the contralateral (non-operated) side, enabling both longitudinal and lateral asymmetry assessment. To account for physiologically plausible positive or negative deviations from reference values and to reduce the impact of outliers, a Gaussian-based normalization approach, which to our knowledge is novel in the context of HD-sEMG analysis, was explored. For each feature of CSI, the deviation from the reference value (either preoperative or contralateral) was transformed using a Gaussian similarity function centered around the reference value. A heuristically defined tolerance zone of ±20% relative to the reference value was used to define the width (i.e., *σ*) of the Gaussian kernel, such that values within this range were considered functionally equivalent. For FPI though the reference value was set to zero, as this should be the ideal state (i.e., constant performance, no fatigue).

##### 2.4.4.3 Global functional index

To obtain a compact summary measure of overall neuromuscular function, a Global Functional Index (GFI) was computed by combining the five functional indices (i.e., AII, EFI, FPI, CSI, SCI). For each session, individual indices were first calculated across all exercises, then z-score normalized within the session to ensure comparability across features and movement types. The GFI was then defined as the equally weighted mean of these normalized values, reflecting an exploratory composite measure of functional muscle quality across dimensions of intensity, spatial focus, fatigue resistance, motor consistency, and pattern similarity.

### 2.5 PROM alignment

To evaluate the clinical relevance and interpretability of the extracted EMG features and derived functional indices, the functional indices were compared with the respective sessions PROM-results. This step aimed to explore whether objective EMG-based metrics reflect patient-experienced function and recovery, thereby supporting the potential applicability of HD-sEMG analysis in personalized rehabilitation assessment.

## 3 Results

**Figure 2:**
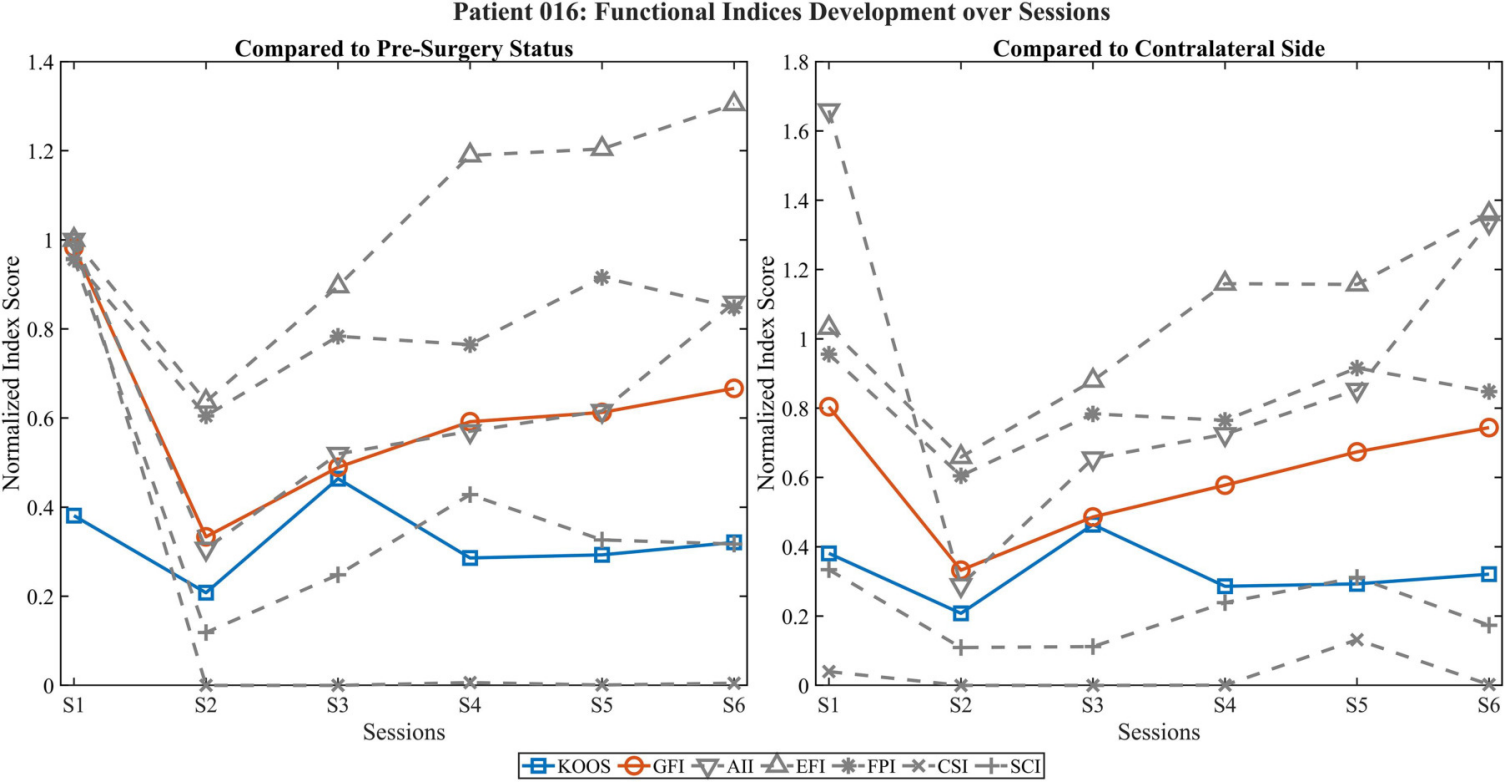
Development of functional EMG indices and KOOS score across postoperative sessions in patient 016. Left panel shows values normalized to the preoperative state; right panel shows normalization relative to the contralateral (non-operated) side. The GFI (red) and the KOOS score (blue) illustrate parallel recovery trends, with an initial decline after surgery (S2) followed by steady improvement across sessions. Gray lines represent individual component indices, including AII, EFI, FPI, CSI, SCI, revealing dimension-specific recovery profiles. Note that despite improving global activation and performance (AII, EFI, FPI), coordination-related indices (CSI, SCI) remain reduced, indicating potential deficits in neuromuscular control.

Figure 2 illustrates the development of functional EMG indices over six postoperative sessions for a representative TKA patient (016), normalized to either the preoperative status (left) or the contralateral side (right). Both the KOOS score and the GFI (red) declined after surgery (S2), followed by a gradual recovery across sessions (S3–S6). Dimension-specific indices (gray) showed varying dynamics: activation- and performance-related indices (AII, EFI, FPI) improved over time, whereas coordination and pattern-based indices (CSI, SCI) remained reduced. Normalization to the contralateral side allowed detection of potential overcompensation (EFI > 1), emphasizing the added value of dual-reference visualization for nuanced interpretation of neuromuscular recovery.

**Figure 3:**
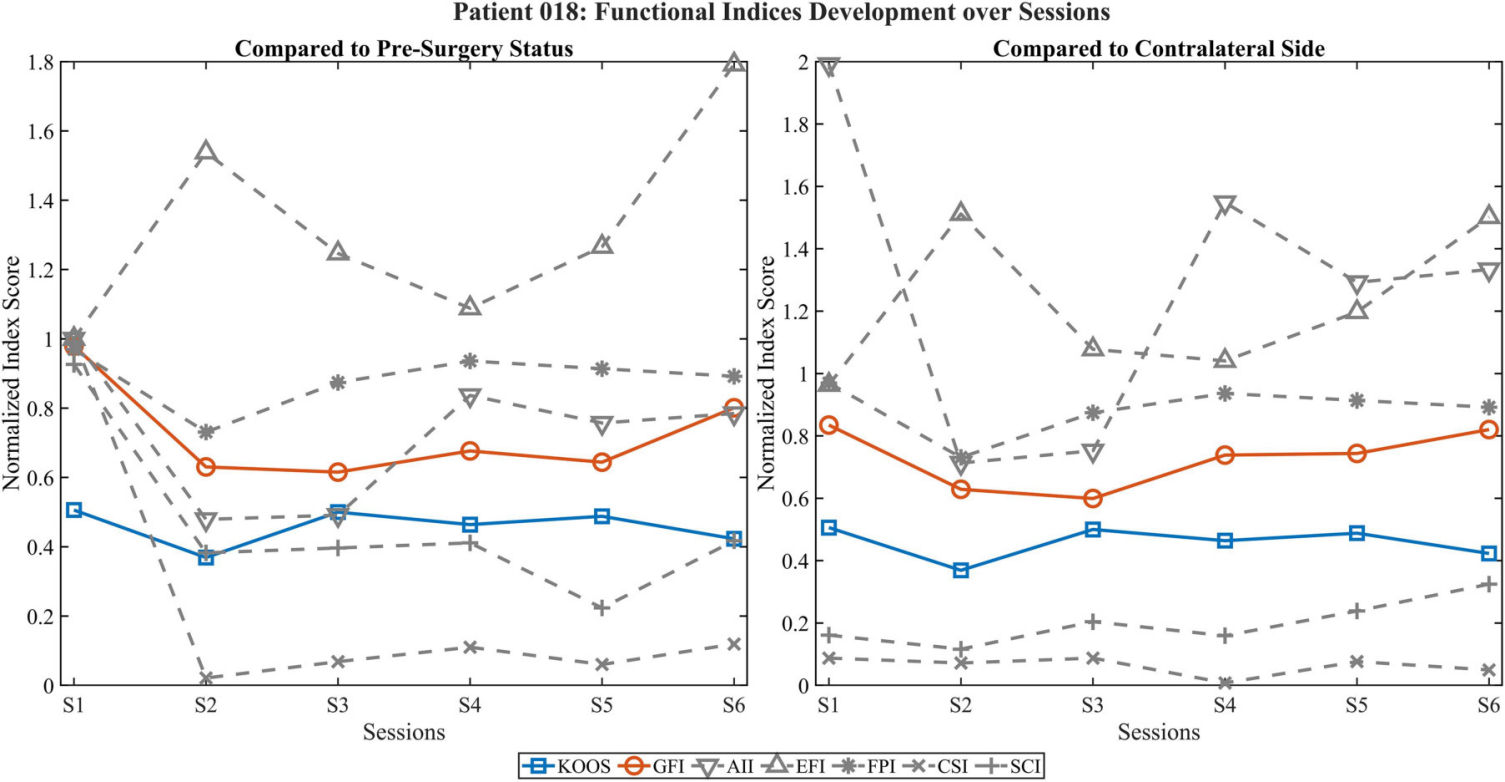
Development of functional EMG indices and KOOS score across postoperative sessions in patient 018. Left panel shows index values normalized to the preoperative state; right panel shows normalization relative to the contralateral side. The KOOS score (blue) remains relatively stable, while the GFI (red) shows a slight improvement across sessions. Activation-based indices (AII, EFI) increase notably, particularly when compared to the contralateral side, suggesting compensatory activation. In contrast, coordination and pattern-based indices (CSI, SCI) remain consistently low, indicating persistent deficits in neuromuscular control despite the observed increase in global activation levels. FPI behaves notably similar to KOOS.

Figure 3 shows another TKA patient (018). While the KOOS score remained relatively stable at a low level, the GFI showed progressive improvement postoperatively. Activation indices increased strongly, particularly in comparison to the contralateral side, suggesting compensatory activation. However, low CSI and SCI values throughout all sessions indicated persistent deficits in motor coordination and spatial consistency. FPI, though offset, shows an almost similar shape as KOOS.

**Figure 4:**
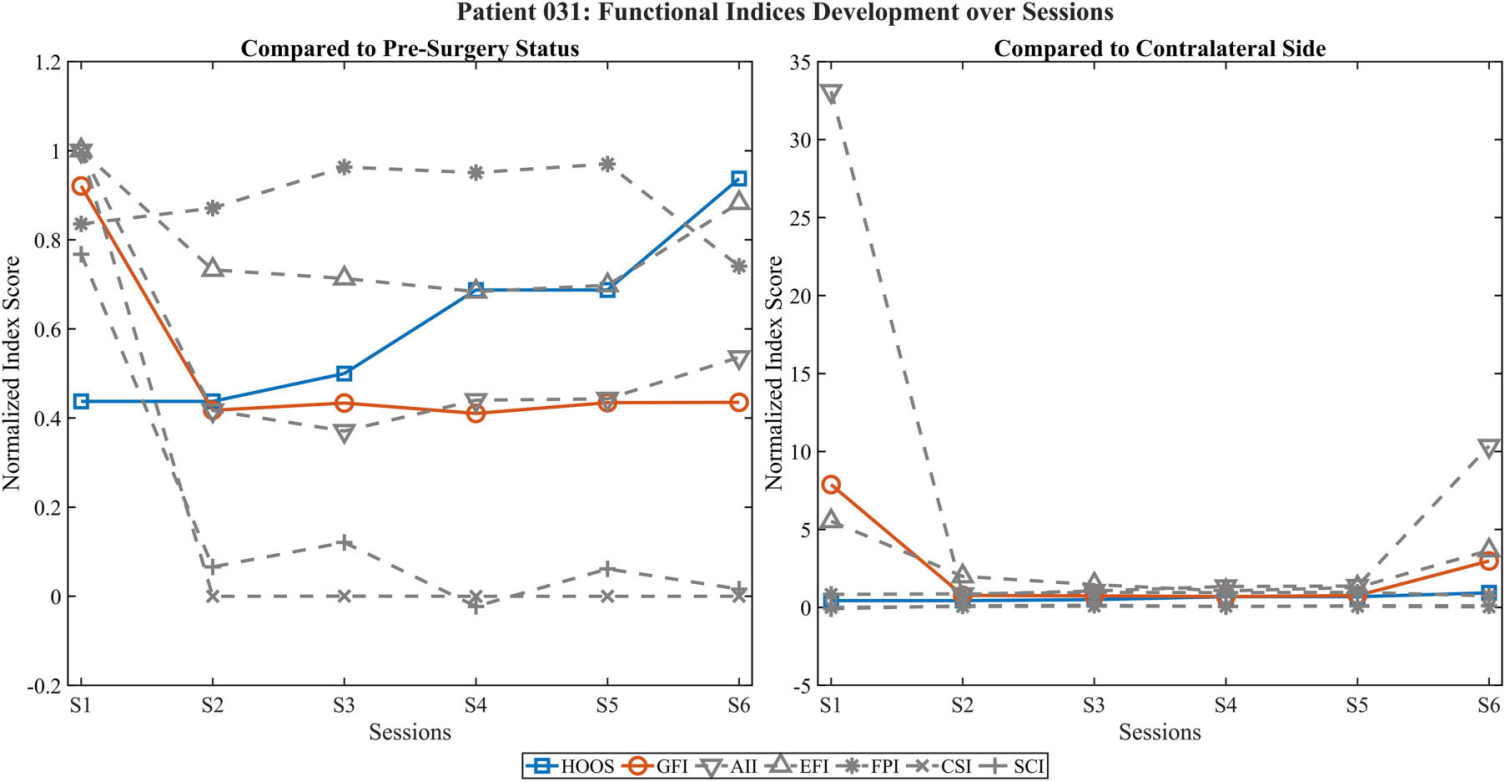
Development of functional EMG indices and HOOS score across postoperative sessions in patient 031. Left panel shows values normalized to the preoperative state; right panel displays normalization relative to the contralateral side. The HOOS score (blue) shows delayed but substantial improvement from session 4 onward, whereas the GFI (red) remains consistently low. Activation-related indices (AII, EFI) show minimal recovery or even exaggerated values due to low contralateral reference activity, while coordination (CSI) and pattern fidelity (SCI) indices stay markedly reduced across all sessions. Also, FPI mildly increases until the last session (S6) where it drops below preoperative state. These findings may reflect compensatory, yet inefficient, activation strategies.

Figure 4 depicts the results for 031, a hip arthroplasty patient. The GFI dropped sharply post-surgery and remained consistently low, whereas the HOOS score showed delayed but continuous improvement. Activation indices (AII, EFI) showed only mild recovery, while CSI and SCI remained minimal. FPI mildly increase until the last session (S6) where it drops below preoperative state. Normalization to the contralateral side revealed exaggerated index values in early sessions (e.g., AII > 30), likely due to low baseline activity on the healthy limb, underscoring the need for robust normalization methods.

## 4 Discussion

The visualization of functional EMG indices over multiple postoperative sessions demonstrated promising alignment with subjective functional recovery, as reflected by the PROMs. Notably, the GFI showed consistent improvements over time in several cases, suggesting that multidimensional EMG-derived metrics can capture relevant aspects of neuromuscular adaptation after joint replacement. The use of both preoperative and contralateral-side normalization enabled complementary perspectives, distinguishing between restitution of baseline function and compensatory activation strategies.

Individual case analyses highlighted the nuanced insights gained through multidimensional evaluation. In patient 016, improvements in activation-related indices (AII, EFI) contrasted with persistently low coordination and pattern-based measures (CSI, SCI), indicating incomplete motor recovery despite overall functional gains. Similarly, patient 018 demonstrated elevated activation levels but poor spatiotemporal control, emphasizing that compensatory activation alone does not equate to functional recovery. In patient 031, representing a hip arthroplasty case, PROM improvements were not mirrored by the GFI or coordination-based indices and extreme values in contralateral-side normalization suggested normalization artifacts due to very low reference activity. These findings underscore the value of integrating multiple feature domains into composite indices such as the GFI to differentiate between true recovery and dysfunctional compensation.

It has to be acknowledged that HD-sEMG decomposition and interpretation are subject to interindividual variability. Factors such as subcutaneous tissue thickness, muscle mass, training status, or patient motivation may influence signal quality and motor unit detectability (46).

Hence, certain limitations of the current index implementation became evident. Manual segmentation was adequate in this setting but remains a barrier to clinical scalability, underscoring the importance of automation in future studies. In some cases, disproportionately high activation values—particularly in AII—led to distorted GFI outcomes. This was evident especially when the contralateral reference values were low. To mitigate this, future index versions may incorporate saturation-like effects through logarithmic transformation or apply threshold-based weighting schemes to reduce outlier influence. These adaptations will require validation on a larger patient cohort before they can be meaningfully implemented.

While the study's longitudinal design included repeated measurements across up to six sessions per patient, these reflect dynamic recovery trajectories rather than stable baselines. Hence, only intra session-consistency-tests and no formal test-retest reliability analysis was conducted. Future studies should incorporate repeated baseline sessions in stable conditions to evaluate inter-session reliability of the proposed indices.

Moreover, some extreme index values (both high and low) suggest the need for further refinement of index construction. As additional datasets become available, statistical approaches such as multivariate regression, principal component analysis, or feature importance analysis (e.g., via random forest models) will be explored to optimize feature selection and weighting. This may allow for more robust, interpretable, and condition-specific indices. Adaptive weighting, outlier-resistant normalization, and clinically grounded interpretation thresholds are potential enhancements to improve sensitivity and generalizability.

### 4.1 Conclusion & future implications

This study demonstrated the feasibility of applying wearable HD-sEMG to assess neuromuscular recovery after total joint replacement in a clinical setting. Electrode array placement and data-acquisition could be performed reliably by a trained assistant physician after a short learning period, suggesting improved accessibility compared to earlier generations of technology. The GFI and its component metrics provided structured, interpretable insights into multidimensional aspects of recovery, capturing changes in activation, coordination, and signal quality. Preliminary trends indicated a potential alignment between EMG-based indices and PROM outcomes, though with subject-specific variability, highlighting the value of objective muscle monitoring alongside subjective measures.

With an aging population and rising rates of orthopedic surgeries, there is a growing need for precise, personalized, and data-driven rehabilitation strategies to improve patient outcomes and reduce long-term healthcare burdens (47–49). HD-sEMG offers a promising modality for non-invasive muscular assessment, and ongoing advances in electrode technology, signal processing, and real-time analytics are likely to further enhance its clinical applicability (17).

Despite promising capabilities, the broader clinical adoption of HD-sEMG is currently limited by several practical hurdles, including data processing complexity, lack of standardized interpretation frameworks, and integration with conventional instrumentation and existing rehabilitation protocols. However, the increasing availability of more accessible and more user-friendly hardware, clinician-oriented software tools (e.g., iSpin, MUedit), and open-source toolboxes (e.g., openhdemg) is expected to lower the entry threshold for clinical researchers and practitioners. Furthermore, as HD-sEMG captures activation patterns that are not accessible through conventional methods, it may contribute to more nuanced understanding of functional compensation, fatigue, or neural drive – especially in longitudinal, therapy-guided applications. Future studies should continue to evaluate the added value of HD-sEMG in complementing established clinical assessments and its potential role in personalized, data-driven rehabilitation strategies.

Current findings are exploratory and limited by sample size and variability in electrode placement. Due to the heterogeneous nature of the population and signal sources, no inferential group statistics were conducted yet. Rather, individual cases were used to illustrate the interpretive potential of the proposed framework. Future work will focus on validating the indices across broader cohorts, refining feature selection and weighting schemes using statistical methods such as multivariate regression and clustering, and exploring HD-sEMG's potential for pre-surgical outcome prediction. Additionally, novel methodological approaches like the Gaussian-based normalization with tolerance zones and the CoG-centered adaptive channel selection will be further investigated in future work to evaluate their robustness, optimize parameter settings and assess their impact on index stability and interpretability across broader datasets. Statistical group comparisons are also subject of future validation studies. Ultimately, this research may support individualized monitoring of recovery trajectories and enable adaptive rehabilitation strategies tailored to patient-specific neuromuscular profiles.

## Data Availability

The raw data supporting the conclusions of this article are available from the corresponding author upon reasonable request. Due to ethical and data protection regulations (GDPR), only pseudonymized data can be shared and within the limits approved by the local ethics committee.
